# ASD2023: towards the integrating landscapes of allosteric knowledgebase

**DOI:** 10.1093/nar/gkad915

**Published:** 2023-10-23

**Authors:** Jixiao He, Xinyi Liu, Chunhao Zhu, Jinyin Zha, Qian Li, Mingzhu Zhao, Jiacheng Wei, Mingyu Li, Chengwei Wu, Junyuan Wang, Yonglai Jiao, Shaobo Ning, Jiamin Zhou, Yue Hong, Yonghui Liu, Hongxi He, Mingyang Zhang, Feiying Chen, Yanxiu Li, Xinheng He, Jing Wu, Shaoyong Lu, Kun Song, Xuefeng Lu, Jian Zhang

**Affiliations:** State Key Laboratory of Medical Genomics, National Research Center for Translational Medicine at Shanghai, Ruijin Hospital, Shanghai Jiao Tong University School of Medicine, Shanghai, China; Medicinal Chemistry and Bioinformatics Center, Shanghai Jiao Tong University School of Medicine, Shanghai 200025, China; State Key Laboratory of Medical Genomics, National Research Center for Translational Medicine at Shanghai, Ruijin Hospital, Shanghai Jiao Tong University School of Medicine, Shanghai, China; Medicinal Chemistry and Bioinformatics Center, Shanghai Jiao Tong University School of Medicine, Shanghai 200025, China; College of Pharmacy, Ningxia Medical University, 1160 Shengli Street, Yinchuan, Ningxia 750004, China; State Key Laboratory of Medical Genomics, National Research Center for Translational Medicine at Shanghai, Ruijin Hospital, Shanghai Jiao Tong University School of Medicine, Shanghai, China; State Key Laboratory of Medical Genomics, National Research Center for Translational Medicine at Shanghai, Ruijin Hospital, Shanghai Jiao Tong University School of Medicine, Shanghai, China; Medicinal Chemistry and Bioinformatics Center, Shanghai Jiao Tong University School of Medicine, Shanghai 200025, China; State Key Laboratory of Medical Genomics, National Research Center for Translational Medicine at Shanghai, Ruijin Hospital, Shanghai Jiao Tong University School of Medicine, Shanghai, China; Medicinal Chemistry and Bioinformatics Center, Shanghai Jiao Tong University School of Medicine, Shanghai 200025, China; State Key Laboratory of Medical Genomics, National Research Center for Translational Medicine at Shanghai, Ruijin Hospital, Shanghai Jiao Tong University School of Medicine, Shanghai, China; Medicinal Chemistry and Bioinformatics Center, Shanghai Jiao Tong University School of Medicine, Shanghai 200025, China; State Key Laboratory of Medical Genomics, National Research Center for Translational Medicine at Shanghai, Ruijin Hospital, Shanghai Jiao Tong University School of Medicine, Shanghai, China; Medicinal Chemistry and Bioinformatics Center, Shanghai Jiao Tong University School of Medicine, Shanghai 200025, China; State Key Laboratory of Medical Genomics, National Research Center for Translational Medicine at Shanghai, Ruijin Hospital, Shanghai Jiao Tong University School of Medicine, Shanghai, China; Medicinal Chemistry and Bioinformatics Center, Shanghai Jiao Tong University School of Medicine, Shanghai 200025, China; Department of Assisted Reproduction, Shanghai Ninth People's Hospital, Shanghai Jiao-Tong University School of Medicine (SJTU-SM), Shanghai 200011, China; State Key Laboratory of Medical Genomics, National Research Center for Translational Medicine at Shanghai, Ruijin Hospital, Shanghai Jiao Tong University School of Medicine, Shanghai, China; Department of Assisted Reproduction, Shanghai Ninth People's Hospital, Shanghai Jiao-Tong University School of Medicine (SJTU-SM), Shanghai 200011, China; State Key Laboratory of Medical Genomics, National Research Center for Translational Medicine at Shanghai, Ruijin Hospital, Shanghai Jiao Tong University School of Medicine, Shanghai, China; Medicinal Chemistry and Bioinformatics Center, Shanghai Jiao Tong University School of Medicine, Shanghai 200025, China; State Key Laboratory of Medical Genomics, National Research Center for Translational Medicine at Shanghai, Ruijin Hospital, Shanghai Jiao Tong University School of Medicine, Shanghai, China; Medicinal Chemistry and Bioinformatics Center, Shanghai Jiao Tong University School of Medicine, Shanghai 200025, China; State Key Laboratory of Medical Genomics, National Research Center for Translational Medicine at Shanghai, Ruijin Hospital, Shanghai Jiao Tong University School of Medicine, Shanghai, China; Medicinal Chemistry and Bioinformatics Center, Shanghai Jiao Tong University School of Medicine, Shanghai 200025, China; Department of Assisted Reproduction, Shanghai Ninth People's Hospital, Shanghai Jiao-Tong University School of Medicine (SJTU-SM), Shanghai 200011, China; State Key Laboratory of Medical Genomics, National Research Center for Translational Medicine at Shanghai, Ruijin Hospital, Shanghai Jiao Tong University School of Medicine, Shanghai, China; Medicinal Chemistry and Bioinformatics Center, Shanghai Jiao Tong University School of Medicine, Shanghai 200025, China; State Key Laboratory of Medical Genomics, National Research Center for Translational Medicine at Shanghai, Ruijin Hospital, Shanghai Jiao Tong University School of Medicine, Shanghai, China; Medicinal Chemistry and Bioinformatics Center, Shanghai Jiao Tong University School of Medicine, Shanghai 200025, China; State Key Laboratory of Medical Genomics, National Research Center for Translational Medicine at Shanghai, Ruijin Hospital, Shanghai Jiao Tong University School of Medicine, Shanghai, China; Department of Assisted Reproduction, Shanghai Ninth People's Hospital, Shanghai Jiao-Tong University School of Medicine (SJTU-SM), Shanghai 200011, China; State Key Laboratory of Medical Genomics, National Research Center for Translational Medicine at Shanghai, Ruijin Hospital, Shanghai Jiao Tong University School of Medicine, Shanghai, China; Medicinal Chemistry and Bioinformatics Center, Shanghai Jiao Tong University School of Medicine, Shanghai 200025, China; State Key Laboratory of Medical Genomics, National Research Center for Translational Medicine at Shanghai, Ruijin Hospital, Shanghai Jiao Tong University School of Medicine, Shanghai, China; Medicinal Chemistry and Bioinformatics Center, Shanghai Jiao Tong University School of Medicine, Shanghai 200025, China; State Key Laboratory of Medical Genomics, National Research Center for Translational Medicine at Shanghai, Ruijin Hospital, Shanghai Jiao Tong University School of Medicine, Shanghai, China; Medicinal Chemistry and Bioinformatics Center, Shanghai Jiao Tong University School of Medicine, Shanghai 200025, China; State Key Laboratory of Medical Genomics, National Research Center for Translational Medicine at Shanghai, Ruijin Hospital, Shanghai Jiao Tong University School of Medicine, Shanghai, China; Medicinal Chemistry and Bioinformatics Center, Shanghai Jiao Tong University School of Medicine, Shanghai 200025, China; State Key Laboratory of Medical Genomics, National Research Center for Translational Medicine at Shanghai, Ruijin Hospital, Shanghai Jiao Tong University School of Medicine, Shanghai, China; Medicinal Chemistry and Bioinformatics Center, Shanghai Jiao Tong University School of Medicine, Shanghai 200025, China; State Key Laboratory of Medical Genomics, National Research Center for Translational Medicine at Shanghai, Ruijin Hospital, Shanghai Jiao Tong University School of Medicine, Shanghai, China; Medicinal Chemistry and Bioinformatics Center, Shanghai Jiao Tong University School of Medicine, Shanghai 200025, China; Nutshell Therapeutics, Shanghai 201210, China; Department of Assisted Reproduction, Shanghai Ninth People's Hospital, Shanghai Jiao-Tong University School of Medicine (SJTU-SM), Shanghai 200011, China; State Key Laboratory of Medical Genomics, National Research Center for Translational Medicine at Shanghai, Ruijin Hospital, Shanghai Jiao Tong University School of Medicine, Shanghai, China; Medicinal Chemistry and Bioinformatics Center, Shanghai Jiao Tong University School of Medicine, Shanghai 200025, China; College of Pharmacy, Ningxia Medical University, 1160 Shengli Street, Yinchuan, Ningxia 750004, China; School of Pharmaceutical Sciences, Zhengzhou University, Zhengzhou 450001, China

## Abstract

Allosteric regulation, induced by perturbations at an allosteric site topographically distinct from the orthosteric site, is one of the most direct and efficient ways to fine-tune macromolecular function. The Allosteric Database (ASD; accessible online at http://mdl.shsmu.edu.cn/ASD) has been systematically developed since 2009 to provide comprehensive information on allosteric regulation. In recent years, allostery has seen sustained growth and wide-ranging applications in life sciences, from basic research to new therapeutics development, while also elucidating emerging obstacles across allosteric research stages. To overcome these challenges and maintain high-quality data center services, novel features were curated in the ASD2023 update: (i) 66 589 potential allosteric sites, covering > 80% of the human proteome and constituting the human allosteric pocketome; (ii) 748 allosteric protein–protein interaction (PPI) modulators with clear mechanisms, aiding protein machine studies and PPI-targeted drug discovery; (iii) ‘Allosteric Hit-to-Lead,’ a pioneering dataset providing panoramic views from 87 well-defined allosteric hits to 6565 leads and (iv) 456 dualsteric modulators for exploring the simultaneous regulation of allosteric and orthosteric sites. Meanwhile, ASD2023 maintains a significant growth of foundational allosteric data. Based on these efforts, the allosteric knowledgebase is progressively evolving towards an integrated landscape, facilitating advancements in allosteric target identification, mechanistic exploration and drug discovery.

## Graphical Abstract

**Graphical Abstract:**
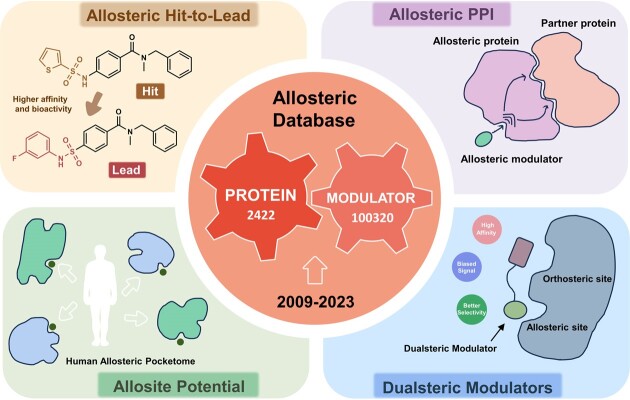

## Introduction

Allosteric regulation, also known as allostery, is a prevalent phenomenon in which the functional site of a macromolecule is fine-tuned by distant allosteric sites in response to various perturbations, including effector binding, point mutations and post-translational modifications (1,2). As an inherent attribute of all dynamic proteins, allosteric regulation bestows exquisite control over diverse biological processes, thus earning it the distinction of ‘the second secret of life’ (3–5). Notably, disease initiation is frequently correlated with aberrations in allosteric regulation, such as myriad allosteric cancer-driving mutations (6). In contrast, targeted manipulation of allosteric drugs towards less conserved allosteric sites provides distinct advantages in terms of selectivity and safety, presenting a pivotal avenue for innovative therapeutic development (7–11).

In recent years, allosteric regulation has been widely applied across various life science branches, revealing an array of significant biological and pathological mechanisms and successfully conquering a wide range of historically intractable drug targets (12–17). However, with the advancement of allosteric strategies, several bottlenecks persist. (i) First, allosteric sites with substantial biological utility remain scarce (18–20). Statistical data indicate that proteins with well-defined allosteric molecule binding sites account for <10% of the total human proteome. A considerable portion of the protein space remains uncharted, indicating the pressing need for further exploration. (ii) Allosteric mechanisms have garnered substantial academic attention, notably in the context of modulating protein–protein interactions (PPIs), a key cornerstone in most cellular processes (21–24). By attaching to distal allosteric sites, effectors induce structural alterations at PPI interfaces, engendering varied and precise functional regulation, such as paclitaxel-stabilizing tubulin PPIs (25) and sotorasib, which inhibit the Ras–Raf interaction (26). Despite their biological significance and therapeutic potential, our understanding of allosteric PPI regulation remains limited, emphasizing the need for suitable informational resources. (iii) In the development of allosteric molecules, it has been observed that the identified allosteric hits frequently display lower binding affinities, grappling with stagnant and unpredictable structure–activity relationships (27,28). With the successful implementation of virtual screening, the study of computer-aided ‘hit-to-lead’ strategies (optimizing hits to improve affinity and bioactivity, generating promising lead molecules) has become a critical topic in allosteric and overall drug discovery (29–31). Despite the ongoing efforts to address this challenge (32–34), the lack of systematic and reliable data hinders the development of universal computational methods. (iv) Moreover, allosteric drugs have certain limitations, including inadequate potency and potential resistance (11,35,36). The combined regulation of orthosteric and allosteric sites offers a viable solution (37). Dualsteric modulators, by linking allosteric and orthosteric pharmacophores, not only confer high affinity and selectivity but also reveal distinct regulatory characteristics such as biased agonism, heralding a new frontier in molecular design (38–41). To facilitate a rational design of these entities, it is necessary to develop a dataset centered on dualsteric modulators.

Since 2009, the Allosteric Database (ASD) has been a central resource providing comprehensive information on allosteric regulation (42–45). Its extensive repository encompasses the structural and genetic characteristics of allosteric proteins and their sites; elaborate details on allosteric modulators and mechanisms; and relevant information on allosteric mutations, drugs, diseases and more. During its 14-year journey, the ASD has been driving the generation of advanced computational tools and resources, facilitating groundbreaking research in allosteric target exploration (46–49), site detection (50–56), modulator discovery (57–59) and evolutionary analyses (60,61). This has led to the identification of first-in-class allosteric molecules for multiple key targets (62–64), earning widespread acclaim within the scientific community (5,65). In ASD2023, to continue providing high-quality data center services and advance the frontiers of research, we have curated several new features: (i) the introduction of the human allosteric pocketome, covering > 80% of the entire human proteome, (ii) the inclusion and elucidation of mechanisms pertaining to 748 known allosteric PPI modulators, (iii) the establishment of panoramic views for allosteric hit-to-lead optimization from 87 well-defined allosteric hits to 6565 leads and (iv) the construction of a detailed dataset comprising 456 dual-steric modulators. ASD has maintained significant growth in traditional allosteric data since its last version (e.g. 500 new allosteric targets, 18000 new allosteric modulators and 19 000 new allosteric interactions). Previously released feature data were further refined and updated. An elaborate description of these improvements is presented in the following section.

## Database growth and statistics

**Figure 1.**
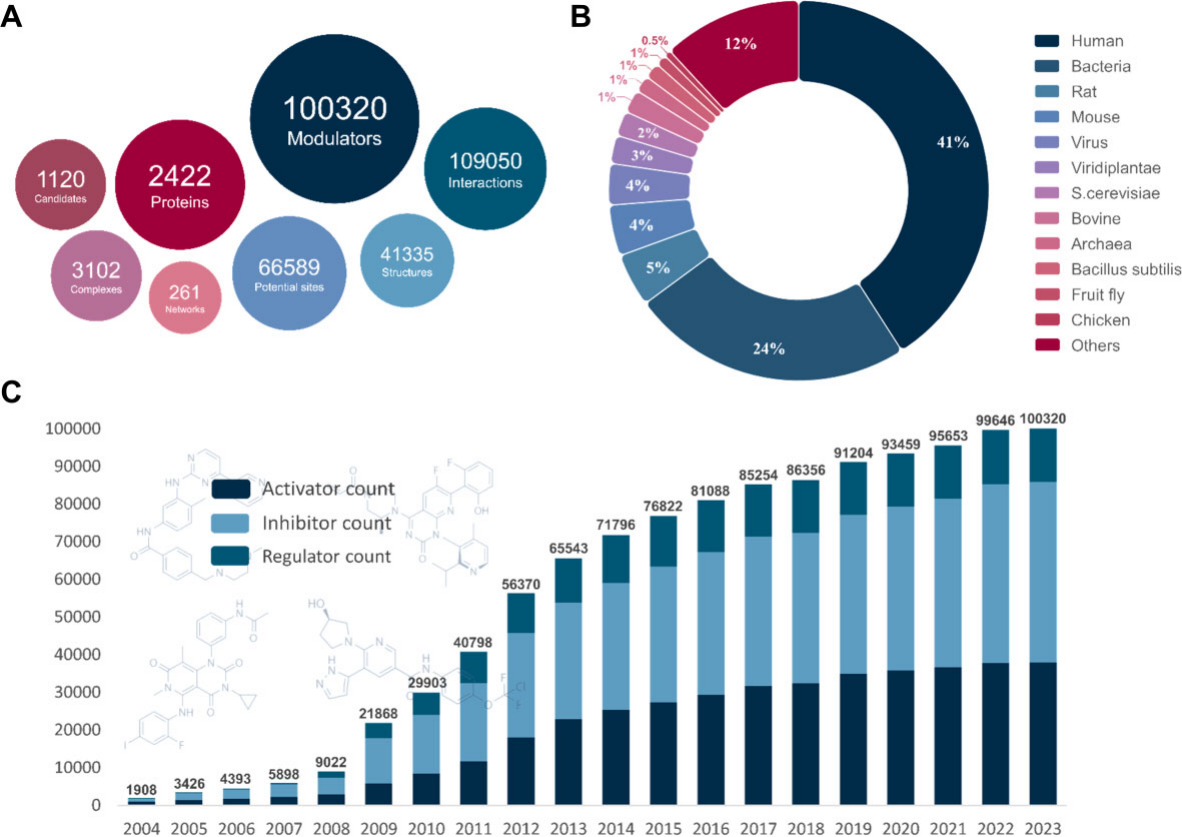
Statistics of foundational allosteric data in ASD2023. (**A**) Overview of ASD2023 data. (**B**) Distribution of allosteric proteins among different organisms. (**C**) Annual growth in the number of allosteric modulators in ASD.

As detailed in Table 1, ASD data consistently demonstrated significant and rapid growth over the past four years, attaining a considerable scale (Figure 1A). The expansion of allosteric proteins continues at a steady and vigorous pace. Currently, ASD contains 2422 allosteric proteins derived from various species, signifying a notable 24.3% increase compared to the previous version (473 new proteins) (45). These proteins are distributed across 27 categories, with kinases, G-protein-coupled receptors (GPCRs),and ion channels occupying prominent proportions, which highlights the pivotal role of allosteric regulation in cellular processes. Hydrolases, oxidoreductases, proteases and transcription factors, among other protein categories, have also undergone substantial growth, suggesting an expanded exploration of allostery across diverse protein domains. Furthermore, these proteins span 426 species (66), with humans, bacteria, rats and mice being the major contributors, accounting for 73.3% of the total, reflecting the profound solicitude of allosteric researchers towards human life and well-being (Figure 1B).

**Table 1.** Data statistics for allosteric proteins and modulators in updated ASD2023

Data category	ASD 2023	ASD 2019
**Number of all proteins** ^a^	2422	1949
Number of kinases	301	249
Number of GPCRs	200	169
Number of ion channels	194	154
Number of hydrolases	196	147
Number of transferases	185	185
Number of oxidoreductases	168	126
Number of transcription factors	152	122
Number of transports	130	120
Number of proteases	128	103
Number of lyases	103	87
Number of other proteins	665	487
**Number of all modulators**	100 320	82 070
Number of activators	38 459	31 376
Number of inhibitors	48 245	37 471
Number of regulators	15 113	14 622
**Number of protein-modulator interactions**	109 050	89 554
**Number of crystal/NMR protein structures**	41 335	26 363
**Number of protein-modulator complex structures**	3102	2453
**Number of Allosteric drug candidates**	1120	538
**Number of potential allosteric sites**	66 589	10 081
**Number of allosteric hit-to-lead series** ^b^	87	0
Number of allosteric hits	87	0
Number of allosteric leads	6565	0
**Statistics of allosteric PPI regulation** ^b^	50	0
Number of allosteric PPI targets	50	0
Number of allosteric PPI modulators	748	0
**Number of dualsteric modulators**	456	0

^a^The classification of allosteric proteins is consistent with ASD 2019.

^b^The majority of the data was already present in ASD, but lacked specific categorization.

Following an incipient rise in 2005–2009, a subsequent explosion from 2010 to 2016, and continuous improvement spanning 2017–2022, the number of allosteric modulators in ASD surpassed a remarkable milestone of 100 000 units (Figure 1C). At present, ASD2023 comprises 100 328 allosteric modulators (an increase of 18 250) distributed among 38 459 activators, 38 459 inhibitors and 15 113 regulators. These modulators primarily consist of small molecules, followed by polypeptides, ions and nucleotides. Accompanying the dual increase in the number of modulators and proteins, the number of allosteric interactions has increased to 109 050.

Alongside the noteworthy expansion of fundamental allosteric data, related features and annotations have been extensively fine-tuned and refreshed, including more crystal/NMR protein structures (from 26 363 to 41 335), protein-modulator complex structures (from 2453 to 3102), drug candidates (from 538 to 1120) and a greater number of potential sites (from 10 081 to 66 589). Moreover, the introduction of entirely new data, including allosteric hit-to-lead series, allosteric PPI regulation and dualsteric modulators, has significantly enriched the allosteric landscape within ASD2023. These specific feature data are elaborated on in the next section.

## New features and functionalities

**Figure 2.**
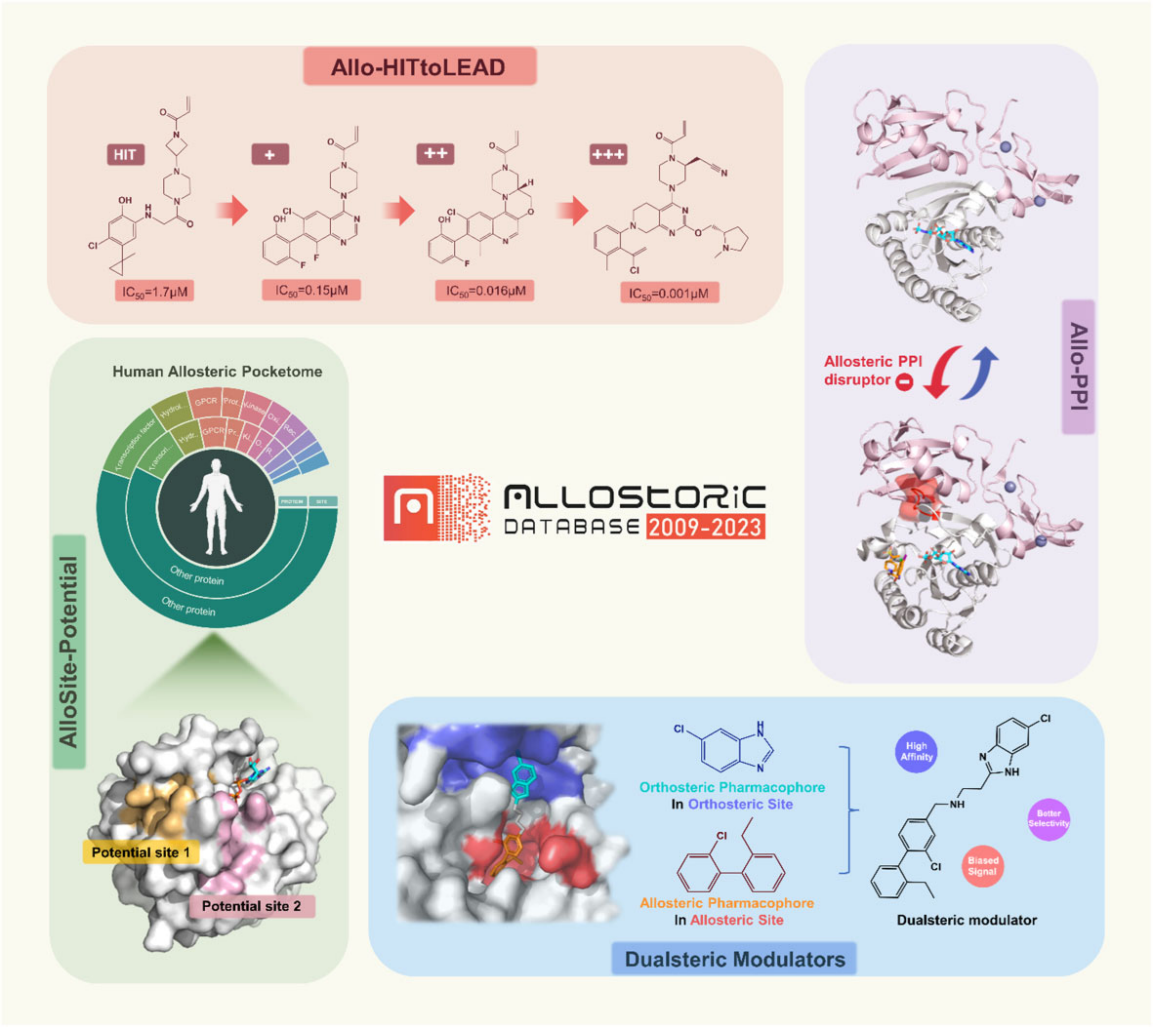
The new features and datasets in ASD2023.

To continue providing data center services and effectively guiding allosteric research, several novel features were curated in ASD2023, as shown in Figure 2. These include ‘Allosite Potential’ for the human allosteric pocketome, ‘Allosteric PPI’ for allosteric PPI regulation, ‘Allosteric Hit-to-Lead’ for hit-to-lead optimization of allosteric modulators, and ‘Dualsteric Modulators’ for reported dualsteric molecules. In addition, ASD2023 has integrated two newly developed allosteric tools, ‘DeepAlloDriver (48)’ and ‘AlloReverse (54),’ which offer novel approaches for identifying allosteric sites, oncogenic allosteric mutations and potential therapeutic targets. New JavaScript codes and 3Dmol.js (67,68), a WebGL-based molecular viewer, have been also incorporated into the website, further enriching user experience.

### Human allosteric pocketome

Allosteric sites, which are topologically distinct from functional sites, are commonly found in most proteins and act as the starting point for allosteric regulation (69). Our previous predictions of potential allosteric sites in existing protein structures has expanded the scope of allosteric research considerably (45). Nevertheless, owing to the scarcity of three-dimensional (3D) protein structure data, their representation in the human proteome remains limited. Recently, the remarkable breakthrough in AI-based protein structure prediction has placed the allosteric exploration across the human proteome within reachable bounds (70,71). In ASD2023, we conducted a comprehensive prediction of the entire human proteome, comprising 20 386 proteins, using AlloSitePro (51). This endeavor culminated in the construction of the ‘Human Allosteric Pocketome,’ a collection of 66 589 potential allosteric sites dispersed across 17 767 proteins, with a protein coverage rate of over 80%. These proteins spanned all protein categories, with GPCR, ion channels and hydrolases having the highest frequency of allosteric sites. Notably, >70% of the proteins revealed allosteric sites with a high potential for molecular targeting and drug development (allosite score > 0.6), highlighting the vast potential of allostery across the human biological network. This updated dataset is accessible through the ‘FEATURES’ menu, under the ‘ALLOSITE-POTENTIAL’ category. Users can refer to ASD2019 for specific instructions regarding data retrieval.

### Allosteric PPI

PPIs, which are integral to numerous cellular processes, present formidable challenges for traditional targeting strategies owing to their typically large and flat interfaces (22). Allosteric modulators, with their distinct regulatory mechanisms, offer promising pharmacological properties and enable precise control over PPIs (23). To allow researchers to efficiently explore and employ comprehensive information of allosteric PPI regulation, we have curated the ‘Allosteric PPI’ in ASD2023, available through the ‘FEATURES’ menu. ‘Allosteric PPI’ is a featured dataset that extensively elucidates the mechanisms of allosteric PPI regulation, including 50 protein-protein interactions and 748 known allosteric modulators. On the first-level page of ‘ALLO-PPI,’ allosteric PPI regulation is classified into two mechanisms: disruption and stabilization. The following list displays the PPIs contained in the dataset along with basic information about the target and partner proteins. Clicking on any PPI field initiates a seamless transition to a secondary page, where detailed information on specific allosteric mechanisms is elucidated. This encompasses information on the PPI crystal structure (apo or holo), residues at the allosteric site and the PPI interface, as well as specific data on the target and partner proteins. Moreover, all corresponding allosteric modulators are listed below, and users can access complete information about each modulator by simply clicking the ‘Show Ligand’ button. The ‘allosteric PPI’ dataset could provide an informative resource for protein machine studies and drug discovery focused on allosteric PPIs.

### Allosteric Hit-to-Lead

Hit-to-lead (H2L) represents a critical stage in early drug discovery, where small-molecule compounds (hits) undergo specific structural optimization to identify promising lead compounds (leads) (29). Utilizing computer- or AI-aided methodologies for the structural optimization of allosteric hits delineates a useful pathway to address the intricate affinity challenges in allosteric molecular design (30). Recognizing the pressing concern of data scarcity in this area, we have established ‘Allosteric Hit-to-Lead’. ‘Allosteric Hit-to-Lead’ is a trailblazing dataset specifically designed for hit-to-lead optimization of allosteric modulators, with all molecular data sourced directly from ASD. It comprises 87 selected allosteric sites, each with well-documented hit-to-lead optimization processes, sourced from 480 high-resolution crystal structures in the ‘core set’ of AsBench (72). For each allosteric site, there is an initial hit and several series of leads. Typically identified using methods such as high-throughput screening (HTS), a hit molecule is the first compound with explicit activity data. The leads are sorted based on activity enhancement in comparison to the hit: a 10-fold increase is LEVEL 1 (+), a 100-fold increase is LEVEL 2 (++), etc. (see Supporting Information). To ensure data reliability, leads are meticulously filtered out if their activity data do not match the hit's record or if binding site information is unclear.

The ‘Allosteric Hit-to-Lead’ dataset amasses 87 hits and 6565 leads, available under ‘ALLO-HITtoLEAD’ in the ‘FEATURES’ menu. In this dataset, users can filter allosteric sites by their leads’ highest optimization level and modulator action type (activator/inhibitor). Clicking on a specific allosteric site opens an interactive page that displays comprehensive hit-to-lead information, including details of the allosteric protein, crystal structure information, and an interactive 3D window for enhanced visualization. By scrolling down the page, users will find a graphic panel that clearly illustrates the step-by-step optimization process from hit to subsequent leads. Further down, exhaustive information about the molecules at every stage is readily accessible. Data regarding negative leads that exhibit no increase in activity will be made available on the website.

**Figure 3.**
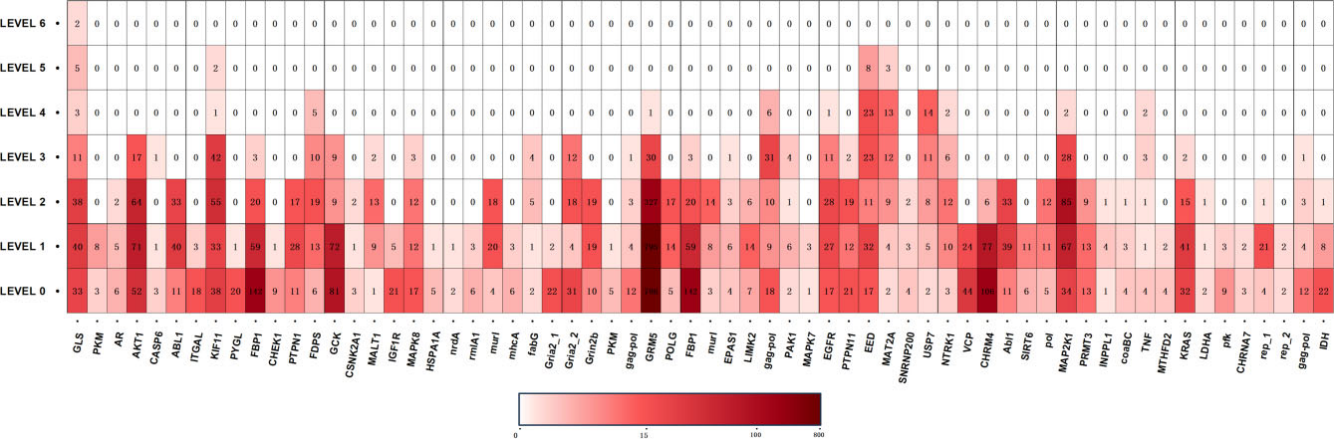
Heatmap displaying the optimization potential of modulators within selected allosteric pockets in the ‘Allosteric Hit-to-Lead’ knowledge map. Detailed information regarding protein and associated residues for each site is available in Supplementary Table S1.

Beyond efforts to optimize the chemical structure, the actual determinant of the druggability of an allosteric modulator lies in the characteristics of its binding sites (27). Hence, identifying allosteric sites with favorable molecular optimization potential (termed ‘optimizability’) is of significant importance in the discovery of allosteric modulators. To expedite the efficient mining and analysis of optimizability traits at allosteric sites, we congregated all relevant available data from the ‘Allosteric Hit-to-Lead’ dataset and created a heat map for the optimizability of selected allosteric pockets (Figure 3). In the knowledge map, an approximate value was assigned for each site's optimizability based on the maximum increase in lead activity (see Supporting Information). The map comprises 133 allosteric sites and their currently achievable optimization levels, spanning over 20 different protein types, ensuring a diverse structural representation.

### Dualsteric modulators

Dualsteric modulators represent an innovative class of chemical ligands that simultaneously bind to both the allosteric and orthosteric sites of a protein, inducing an allosteric effect while also exerting functional responses or competing for substrate binding (38). This distinctive dual-binding mechanism grants them superior affinity and selectivity along with the potential for signal biasing, which greatly ameliorates the deficiencies of allosteric molecules (39,73). Thus, dual-steric modulators have emerged as a novel frontier in molecular design and have received increasing attention. Building upon the existing allosteric knowledgebase, we present ‘Dualsteric Modulators’ as the inaugural dataset centered on these exceptional molecules. This dataset offers detailed insights into design rationale and binding mechanism of 456 dualsteric modulators, readily available via the ‘FEATURE’ menu. Within the ‘Dualsteric Modulators’ interface, users can access detailed information pages by clicking on the molecule of interest. They were first presented with the target protein information, accompanied by an interactive 3D window. The design rationale of the dualsteric modulator is displayed in the following visualization panel, which shows the structure and related information of the original orthosteric/allosteric molecules. Other dualsteric modulators that bind to the same protein and site are listed below. In addition to the 472 dualsteric modulators, the collection also includes 28 dual-allosteric modulators that bind to two allosteric sites, laying the foundation for exploring the regulation of dual sites at the same time.

## Conclusion and future directions

The ASD is a comprehensive and integrated allosteric data platform that reflects the remarkable progress achieved in allosteric research over the past 14 years (see Supplementary Figure S1). Building upon previous versions, the ASD maintains an increase of over 20% in each category of foundational allosteric data encompassing proteins, molecules, interactions, sites, and protein structures. Furthermore, to overcome the bottlenecks encountered at different stages of allosteric research, we introduced innovative datasets tailored to the human allosteric pocketome, allosteric PPI regulation, hit-to-lead molecule optimization, and dualsteric modulators. Additionally, multiple newly developed allosteric tools have been integrated into ASD and are easy to use. Based on these endeavors, our allosteric knowledge base is steadily progressing towards an integrated landscape.

The primary objective of ASD is to provide first-rate allosteric information resources and central services to a wide audience. In the future, our emphasis will be on gathering and integrating high-quality allosteric data while expanding their breadth and depth. We will also actively seek community feedback to enhance the database architecture and tools to ensure optimal user interaction. Finally, ASD will steadily concentrate on cutting-edge allosteric research, offering copious data to improve our understanding of allosteric origins, genetics, pathogenesis, and novel mechanism-driven drug discovery.

## Supplementary Material

gkad915_Supplemental_File

## Data Availability

The database is freely available at: http://mdl.shsmu.edu.cn/ASD.
