# Supplementary material for: ASD2023: towards the integrating landscapes of allosteric knowledgebase
Source: Nucleic Acids Res. 2023 Oct 23;52(D1):D376–83. doi: 10.1093/nar/gkad915 (PMC10767950; doi:10.1093/nar/gkad915)
Supplement: gkad915_Supplemental_File [file gkad915_supplemental_file.docx]

**SUPPLEMENTARY DATA**

**A**

**ASD2023: towards the integrating landscapes of allosteric knowledgebase**

Jixiao He^1,2,||^, Xinyi Liu^1,2,||^, Chunhao Zhu^3,||^, Jinyin Zha^1,||^, Qian Li^1,2,||^, Mingzhu Zhao^1,2^, Jiacheng Wei^1,2^, Mingyu Li^1,2^, Chengwei Wu^1,2,4^, Junyuan Wang^1,4^, Yonglai Jiao^1,2^, Shaobo Ning^1,2^, Jiamin Zhou^1,2,4^, Yue Hong^1,2^, Yonghui Liu^1,2^, Hongxi He^1,4^, Mingyang Zhang^1,2^, Feiying Chen^1,2^, Yanxiu Li^1,2^, Xinheng He^1,2^, Jing Wu^1,2^, Shaoyong Lu^1,2^, Kun Song^5^, Xuefeng Lu^4,*^ and Jian Zhang^1,2,3,6,*^

^1^ State Key Laboratory of Medical Genomics, National Research Center for Translational Medicine at Shanghai, Ruijin Hospital, Shanghai Jiao Tong University School of Medicine, Shanghai, China.

^2^ Medicinal Chemistry and Bioinformatics Center, Shanghai Jiao Tong University School of Medicine, Shanghai, 200025, China.

^3^ College of Pharmacy, Ningxia Medical University, 1160 Shengli Street, Yinchuan, Ningxia 750004, China.

^4^ Department of Assisted Reproduction, Shanghai Ninth People's Hospital, Shanghai Jiao-Tong University School of Medicine (SJTU-SM), Shanghai 200011, China.

^5^ Nutshell Therapeutics, Shanghai, 201210, China.

^6^ School of Pharmaceutical Sciences, Zhengzhou University, Zhengzhou, 450001, China.

**Key Words:** ASD, allostery, allosteric protein, allosteric modulator, allosteric site

^||^ The authors equally contribute to this work

***To whom correspondence should be addressed:**

Jian Zhang

Email: [Jian.zhang@sjtu.edu.cn](mailto:Jian.zhang@sjtu.edu.cn)

Tel: +86-21-63846590

Fax: +86-21-64154900

Xuefeng Lu

Email: [xuefenglu163@163.com](mailto:xuefenglu163@163.com)

Tel: +86-21-63846590

Fax: +86-21-64154900

**MATERIALS AND METHODS**

**Allosite-Potential V2**

In the Allosite-Potential V2, foundational structural data primarily stems from the Protein Data Bank (PDB, https://www.pdbus.org/) (1) and the Alphafold Protein Structure Database (<https://alphafold.com/>) (2). After filtration and extraction from the PDB, 4,486 human proteins were obtained with 41,792 X-ray structures, while 1,714 proteins had 3,489 NMR structures. For the collection of 23,391 Alphafold-derived structures (20,505 human proteins), B-factor values were initially estimated using the ‘ResQ’ method (3). Next, the ‘Phenix’ tool was employed to remove their low-confidence residues (pLDDT < 70), converted error B-value estimates, and refined the processed model (4). Subsequently, leveraging our prior-developed AllositePro methodology, potential allosteric sites in all aforementioned structures were predicted with a threshold of AlloSite score ≥ 0.5 (5). If the score is less than 0.5, a lower threshold of AlloSite score ≥ 0.2 is reconsidered. For the same protein, the experimentally validated structure with the highest-scoring site is prioritized for display on the webpage. Finally, we constructed a human allosteric pocketome encompassing 66,589 potential allosteric sites throughout the human proteome.

**Validation of Allosite-Potential V2**

Allosite-Potential V2 is a dataset of allosteric sites in human proteome predicted by AllositePro. Since AllositePro was only validated on a small test set, a further evaluation is required. To do so, we first extracted all PDB structures with reported allosteric sites from Allosite-Potential. Then, we examined whether these sites have been successfully predicted in Allosite-Potential. The examination is performed as followed. For each reported site, we calculated its maximum ratio of shared residues with predicted allosteric sites of the same PDB structure. If the maximum ratio is above 40%, the reported site is regarded as a successful prediction. Consequently, among the reported 1409 human allosteric sites, we have identified 833 sites that have re-emerged, constituting a proportion of 59.4%, consistent with prior reports.

**Allosteric PPI**

The ‘Allosteric PPI’ dataset primarily collates data from distinguished scientific publications and recognized public online resources: e.q. PDB, Interactome INSIDER (6), and PubChem (7). Using search terms like ‘Alloster*’, ‘PPI’, and ‘protein-protein interaction’, 425 articles related to allosteric PPI regulation were identified from the extant ASD literature repository and databases such as PubMed. Subsequently, a specialized team of researchers meticulously extracted and logged data on allosteric proteins, partner entities, and allosteric modulators from these studies, leading to the collection of 50 protein-protein interaction pairs accompanied by 456 related allosteric PPI modulators. For each allosteric PPI pair, crystal structures, interfaces residues, and allosteric site residues were obtained from PDB and Interactome INSIDER. When absent from these resources, data were manually retrieved from the relevant literature and confirmed. With the utilization of PyMOL, residues around allosteric modulators were extracted via the ‘Select’ command (cutoff = 5), while those at the PPI interface were pinpointed using ‘interfaceResidue’ (cutoff set at 20). Modulator details, most archived in ASD already, including chemical structure, mechanism of action, and bioactivity, were enriched from source articles, PubChem, and Drugbank (8).

**Allosteric Hit-to-Lead**

‘Allosteric Hit-to-Lead’ is an innovative dataset designed for structural optimization of allosteric modulators and the optimizing potential of allosteric sites, with all molecular data sourced directly from ASD. The construction of this dataset involved a multi-step approach: (i) Within our literature repository, molecular progressions was traced for 480 allosteric sites, each equipped with high-resolution crystal structures, sourcing from the ‘core set’ of AsBench (9). This exploration confirmed 150 allosteric sites with specific exogenous hit (the first compound with explicit activity data) and methodically documented structural optimization process. (ii) All molecules corresponding to these 150 allosteric proteins were extracted from ASD. Subsequent filtration was conducted based on the hit's mechanism of action, activity data type, and binding site. During this procedure, 87 sites were pinpointed to have a distinct hit-to-lead optimization pathway, refining the preliminary pool of 22,343 molecules down to a curated set of 6,565 lead compounds. (iii) Activity data of these leads underwent a comparative analysis with the hit molecule. If there was an enhancement in activity, the lead molecules were systematically classified and displayed on the webpage: a 10-fold (10-99 times) increase is designated as LEVEL 1 (+), a 100-fold (100-999 times) increase as LEVEL 2 (++), and so forth. Conversely, compounds without activity enhancement were incorporated into a negative dataset, serving as controls. (iv) The optimization potential of molecules within each allosteric site is simply evaluated by the maximum optimization level: sites demonstrating an increase less than 10-fold (i.e., 0-9 times) garner a 20% rating, while sites with a LEVEL 1 enhancement are given 30%, and so on. Ultimately, by interlinking allosteric sites with their respective optimizability, a comprehensive knowledge map was formulated.

**Dualsteric Modulators**

Consistent with existing allosteric data, foundational information for dualsteric modulators is chiefly derived from PubMed via the keywords of ‘dualster* OR (alloster* AND bitopic)’. Then, information regarding targets, chemical structures, biological activities, and binding sites of dualsteric modulators was manually extracted from published articles. In this dataset, the dualsteric modulator’s two primary facets, the orthosteric and allosteric pharmacophore, are individually explored with comprehensive detail. Tracing back to the original literature, the source molecules for each orthosteric and allosteric pharmacophore, along with their basic information, were pinpointed. For dualsteric modulators lacking identified source molecules, their orthosteric/allosteric pharmacophores were documented as an alternative. Moreover, the orthosteric and allosteric binding sites for each dualsteric modulator are meticulously noted and showcased within a 3D visualization panel. Our curated dataset interlinks the 456 dualsteric modulators, drawing connections rooted in their 21 specific targets and binding sites.

**FIGURE**

**Figure S1. Comprehensive overview of the functions and capabilities of ASD**

**
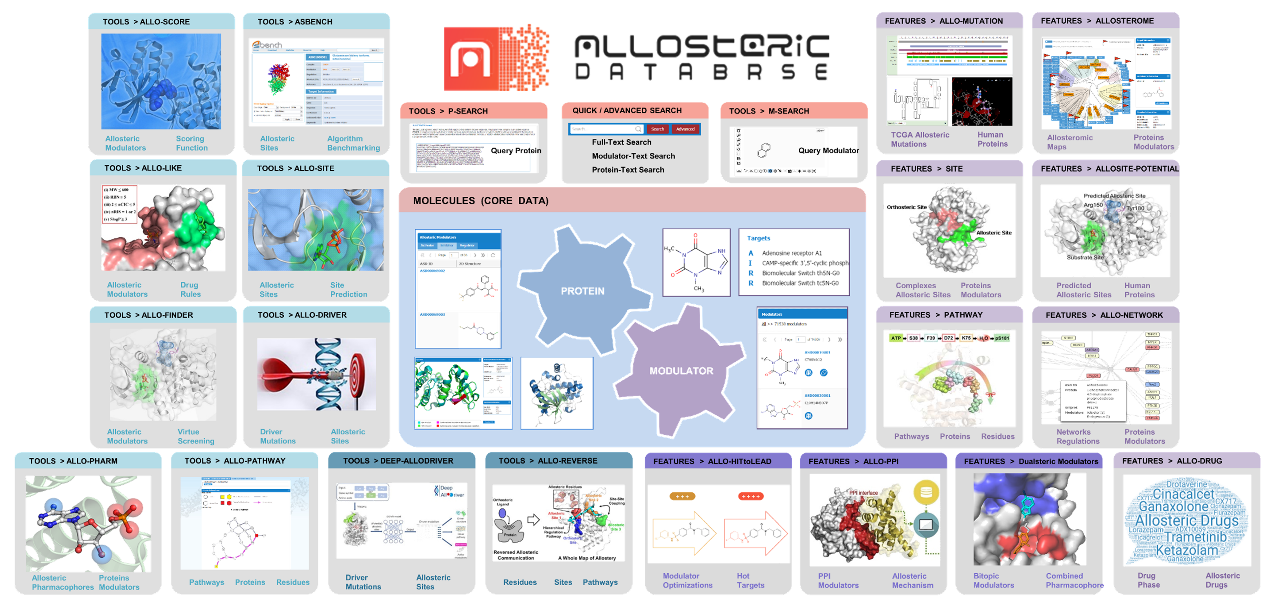
**

**TABLE**

**Table S1. Detailed information on selected allosteric sites in Figure 3**

| Target site (Gene)^a^ | Target_name | Uniprot | PDB ID | Allosite residues | site potential |
| --- | --- | --- | --- | --- | --- |
| GLS | Glutaminase kidney isoform, mitochondrial | O94925 | 3UO9 | Chain A:LYS320,LEU321,PHE322,LEU323,ASN324,GLU325,ASP327,TYR394 | 80% |
| PKM | Pyruvate kinase | P14618 | 4G1N | Chain A:PHE26,MET30,LEU353,ASP354,ILE389,TYR390,GLN393,LEU394,GLU397 | 30% |
| AR | Androgen receptor | P10275 | 2YLO | Chain A:PRO723,GLY724,PHE725,ARG726,ASN727,PHE826,GLU829,LEU830,ASN833,TYR834 | 40% |
| AKT1 | RAC-alpha serine/threonine-protein kinase | P31749 | 3O96 | Chain A:TRP80,THR82,VAL83,ILE84,GLU85,SER205,LEU210,THR211,LEU264,LYS268,VAL271,TYR272,ARG273,ASP274,ILE290,THR291,ASP292,CYS296 | 50% |
| CASP6 | Caspase-6 | P55212 | 4NBN | Chain A:VAL197,TYR198,THR199,LEU200,PRO201,GLU214 | 50% |
| ABL1 | Tyrosine-protein kinase ABL1 | P00519 | 5MO4 | Chain A:TYR361,ALA356,PRO484,GLU481,LEU359,TRP449,PHE512,PRO480,CYS483,THR453,ARG351,ASN355,LEU448,THR364,LEU360,MET456,GLY482,TYR454,LEU529,LEU447,VAL487,VAL525,ALA363,MET491,ALA452,ILE521,ILE451 | 40% |
| ITGAL | Integrin alpha-L | P20701 | 3M6F | Chain A:VAL130,PHE134,VAL157,LEU161,TYR166,THR231,VAL233,ILE235,ILE255,TYR257,ILE259,LYS287,LEU302,LYS305,ILE306 | 30% |
| KIF11 | Kinesin-like protein KIF11 | P52732 | 3ZCW | Chain A:TYR104,LEU266,SER269,GLU270,ASN287,ILE288,ASN289,LEU292,LEU293,LEU295,GLY296,ARG297,ILE299,THR300,ILE332,TYR352,ALA353,ARG355,ALA356 | 70% |
| PYGL | Glycogen phosphorylase | P06737 | 3CEH | Chain A:TRP67,ILE68,GLN71,GLN72,TYR75,LYS191,ARG193,ASP227 | 30% |
| FBP1 | Fructose-1,6-bisphosphatase 1 | P09467 | 2JJK | Chain A:VAL17,MET18,GLU20,GLY21,ARG22,ALA24,GLY26,THR27,GLY28,GLU29,LEU30,THR31,LEU34,MET177; Chain C:VAL17,MET18,GLY21,ARG22,ALA24,GLY26,THR27,GLY28,GLU29,LEU30,THR31,LEU34,MET177 | 50% |
| CHEK1 | Serine/threonine-protein kinase Chk1 | O14757 | 3F9N | Chain A:PHE93,ILE96,ASP99,PRO133,ALA200,GLY204,GLU205,LEU206,PRO207,TRP208,ASP209,GLU217 | 30% |
| PTPN1 | Tyrosine-protein phosphatase non-receptor type 1 | P18031 | 1T49 | Chain A:ALA189,LEU192,ASN193,PHE196,LYS197,GLU200,GLU276,GLY277,LYS279,PHE280,ILE281,MET282 | 40% |
| FDPS | Farnesyl pyrophosphate synthase | P14324 | 3N1V | Chain F:TYR10,LYS57,ASN59,ARG60,THR63,SER205,PHE206,PHE239,LEU344,LYS347 | 60% |
| GCK | Glucokinase | P35557 | 3H1V | Chain X:TYR61,VAL62,ARG63,SER64,THR65,GLU96,GLY97,GLN98,ILE159,MET210,ILE211,TYR214,TYR215,HIS218,CYS220,MET235,LEU451,VAL452,VAL455 | 50% |
| CSNK2A1 | Casein kinase II | P68400 | 3H30 | Chain A:TYR39,GLN40,LEU41,VAL67,ILE69,VAL101,ASP103,PRO104,ALA110 | 60% |
| MALT1 | Mucosa-associated lymphoid tissue lymphoma translocation protein 1 | Q9UDY8 | 4I1R | Chain A:VAL344,ALA345,LEU346,LYS379,VAL381,ALA394,GLU397,PHE398,LEU400,LEU401,ARG576,TRP580,LEU715,MET717 | 50% |
| IGF1R | Insulin-like growth factor 1 receptor | P08069 | 3LW0 | Chain A:LYS1033,GLU1050,ALA1051,MET1054,VAL1062,VAL1063,LEU1065,VAL1077,MET1079,HIS1133,ARG1134,ILE1151,GLY1152,ASP1153,LEU1174,PHE1189 | 30% |
| MAPK8 | Mitogen-activated protein kinase 8 | P45983 | 3O2M | Chain A:THR178,PHE180,ILE197,LEU198,GLY199,TYR230,ILE231,TRP234,GLN253,THR255,VAL256,TYR259; Chain B:PRO184,THR255 | 50% |
| HSPA1A | Heat shock 70 kDa protein 1A | P0DMV8 | 4IO8 | Chain A:TYR15,ASN35,THR37,GLY202,GLY230,GLU268,LYS271,ARG272,SER275,GLY339,SER340,ARG342,ILE343,ASP366 | 30% |
| nrdA | Ribonucleotide reductase | P00452 | 2R1R | Chain A:ASP232,SER233,LEU234,ARG262,ILE268,ARG269,HIS275,THR276; Chain B:SER249 | 30% |
| rmlA1 | Glucose-1-phosphate thymidylyltransferase 1 | P37744 | 1H5S | Chain A:LEU46,TYR115,GLY116,HIS117,ASP118,LYS250,VAL251,SER252,GLU256,ILE257,ARG260; Chain D:GLY219,ARG220,GLY221 | 30% |
| murI | Glutamate racemase | P22634 | 2JFN | Chain A:LEU100,ARG104,VAL112,GLY113,VAL114,VAL115,PRO116,ALA117,LYS119,PRO120,PHE151,SER227,ALA230,ILE231,ARG233,ARG234 | 40% |
| mhcA | Myosin-2 heavy chain | P08799 | 2JHR | Chain A:LYS265,ALA420,LYS423,ALA424,GLY427,ARG428,LEU431,ASP590,LEU592,ILE617,ALA618,SER619,ARG620 | 30% |
| fabG | 3-oxoacyl-[acyl-carrier-protein] reductase FabG | O54438 | 4BO2 | Chain A:TRP106,PHE107,VAL110,ASN111,LEU114,ALA156,ALA159,GLY160,GLY163,PHE164 | 50% |
| Gria2_1 | Glutamate receptor 2 | P19491 | 4N07 | Chain A:LYS104,PRO105,PHE106,MET107,SER108,LEU239,SER242,LEU247 | 30% |
| Gria2_2 | Glutamate receptor 2 | P19491 | 3LSF | Chain E:PRO105,PHE106,MET107,SER108,SER242,LEU247,ASP248,LYS251 | 50% |
| Grin2b | Glutamate receptor ionotropic, NMDA 2B | Q00960 | 3QEL | Chain A:TYR109,THR110,ARG115,SER132,ILE133,LEU135; | 40% |
| PKM | Pyruvate kinase | P11974 | 3N25 | Chain A:ARG42,ASN43,ASN69,ARG105,HIS463,TYR465,GLY467,ILE468,PHE469,PRO470 | 30% |
| gag-pol | HIV-1 capsid protein | P12497 | 4AHS | Chain A:GLY106,ARG107,TRP108,PRO109,ILE204,ILE208; Chain B:TYR83,HIS185,GLY197,GLU198,VAL201 | 50% |
| GRM5 | Metabotropic glutamate receptor 5 | P41594 | 4OO9 | Chain A:GLY624,ILE625,ILE651,SER654,PRO655,SER658,TYR659,VAL740,PRO743,LEU744,ASN747,ILE751,TRP785,PHE788,MET802,SER805,VAL806,SER809,ALA810,ALA813 | 60% |
| POLG | Genome polyprotein | Q9WMX2 | 4NLD | Chain A:LEU392,ALA393,ALA395,ALA396,THR399,ALA400,ILE424,LEU425,HIS428,PHE429,LEU492,GLY493,VAL494,PRO495,TRP500,ARG503 | 40% |
| FBP1 | Fructose-1,6-bisphosphatase 1 | P00636 | 1KZ8 | Chain A:VAL17,GLN20,GLY21,ALA24,GLY26,THR27,GLY28,GLU29,MET30,THR31,LYS112,TYR113,ARG140,VAL160,MET177 | 50% |
| murI | Glutamate racemase | Q9ZLT0 | 2W4I | Chain A:PRO38,THR41,SER143,VAL146,PRO147,GLU150,GLU151 | 40% |
| EPAS1 | Endothelial PAS domain-containing protein 1 | Q99814 | 3H7W | Chain A:SER246,HIS248,MET252,ALA277,TYR281,MET289,SER292,HIS293,LEU296,VAL302,SER304,TYR307,MET309,THR321,ILE337,CYS339,ASN341 | 50% |
| LIMK2 | LIM domain kinase 2 | P53671 | 4TPT | Chain A:VAL467,THR405,LEU472,MET380,ILE476,ARG381,ILE447,GLY471,VAL388,ALA468,PHE342,ARG474,GLU361,ILE363,LEU442,LEU403,ASP469,ARG364,PHE391,LEU362,LYS360,PHE470,HIS449,LYS390,LEU383,LEU389 | 40% |
| gag-pol | Gag-Pol polyprotein | P0C6F2 | 4NYF | Chain B:GLU96,ALA129,GLY94,ALA124,GLN95,TYR99,THR125,ALA133,ALA128,LEU102,ALA98,TRP132 | 40% |
| PAK1 | Serine/threonine-protein kinase PAK 1 | Q13153 | 4ZLO | Chain A:VAL328,ARG320,ASN329,THR406,ASN322,GLU315,PHE379,VAL385,GLN384,MET319,ASN383,LYS323,ASN314,HIS387,LEU380,ILE327,ASP407,VAL318,LEU405 | 50% |
| MAPK7 | Mitogen-activated protein kinase 7 | Q13164 | 4ZSG | Chain A:GLY67,VAL135,ALA65,ILE117,THR99,THR94,LEU100,VAL68,LEU106,ASP200,LEU137,ALA96,LYS84,LEU103,ILE86,TYR66,ASN95,ARG98,ALA89,GLU102 | 30% |
| EGFR | Epidermal growth factor receptor | P00533 | 5D41 | Chain A:LYS745,TYR727,VAL769,LEU777,PHE856,GLU758,VAL726,VAL774,LEU760,MET766,ILE780,ARG776,LEU858,GLY857,ILE744,THR854,ALA743,LEU747,PHE723,LEU788,ILE853,CYS775,ILE789,ALA763,GLU762,ILE759,MET790,ASP855 | 60% |
| PTPN11 | Tyrosine-protein phosphatase non-receptor type 11 | Q06124 | 5EHR | Chain B:GLU250,PRO491,THR218,LEU254,ARG111,THR219,SER109,GLN257,TYR511,GLN495,THR253,GLN255,ASN217,GLY115,PHE113,PHE251,PRO107,GLU110,THR108,LYS492,GLY246,GLU249,LEU216,HIS114,TRP112,ASP489 | 50% |
| EED | Polycomb protein EED | O75530 | 5U5T | Chain B:GLN415,TYR365,GLY98,LYS211,MET256,SER241,ASN194,LEU240,PHE97,MET366,ARG367,ASP310,ARG414,TYR148,GLU238,ALA192,LEU96,ILE193,CYS311 | 70% |
| MAT2A | S-adenosylmethionine synthase isoform type-2 | P31153 | 5UGH | Chain C:PHE18,SER331,GLN317,LEU315,GLY275,THR17,TYR335,GLY16,PHE20,GLN190,PHE139,ALA276,LEU19,GLU342,TRP274,VAL316,PHE333,ILE332,ARG313,GLY273,GLU15 | 70% |
| SNRNP200 | U5 small nuclear ribonucleoprotein 200 kDa helicase | O75643 | 5URJ | Chain A:PRO1680,PHE1717,PRO1257,LYS1711,PHE1254,PHE1255,LEU1198,GLY1460,ASP1678,ILE1681,THR1253,ILE1193,LEU1722,TYR1679,ASP1712,VAL1724,HIS1235,PRO1723,PHE1713,TYR1682,SER1196,LYS1716,GLU1237,SER1709,PHE1714,LYS1710,THR1197,VAL1256,THR1666 | 40% |
| USP7 | Ubiquitin carboxyl-terminal hydrolase 7 | Q93009 | 5N9T | Chain A:LYS420,GLN351,GLY458,ALA513,ASP295,HIS294,VAL296,TYR514,ASN460,ILE419,HIS461,PHE409,LEU406,ILE421,ASP459,GLN297,TYR224,ASN512,GLN405,TYR465,HIS456,MET407,GLY462,MET410,CYS223,ARG408 | 60% |
| NTRK1 | High affinity nerve growth factor receptor | P04629 | 6D20 | Chain A:LEU516,TRP514,LYS513,LEU526,GLU515,LEU512,PHE525 | 60% |
| VCP | Transitional endoplasmic reticulum ATPase | P55072 | 5FTJ | Chain A:THR509,GLY513,ALA537,THR613,VAL493,CYS535,LYS512,GLU534,ASN616,VAL497,VAL573,GLU498,PRO496,PRO510,PHE618,LEU492,LYS615,CYS572,GLN494,SER511,VAL617,PRO571,LYS614,GLN536 | 30% |
| CHRM4 | Muscarinic acetylcholine receptor M4 | P08173 | 5DSG | Chain A:ASN423,THR196,TYR89,GLN188,ILE187,TRP108,TYR416,LEU190,VAL420,ASP432,TRP435,SER191,LEU109,CYS185,SER436,PHE186,TYR92,TYR113,TYR439 | 40% |
| Abl1 | Tyrosine-protein kinase ABL1 | P00520 | 3K5V | Chain A:ALA356,LEU359,LEU360,ALA363,LEU448,ILE451,ALA452,TYR454,GLU481,GLY482,CYS483,PRO484,VAL487,PHE512,ILE521,VAL525,LEU529 | 40% |
| SIRT6 | NAD-dependent protein deacetylase sirtuin-6 | Q8N6T7 | 5Y2F | Chain A:GLU85,ASN2,TRP69,ALA5,PRO60,MET155,PHE84,VAL68,MET134,VAL151,LYS79,THR83,PRO78,PHE80,VAL1,GLY153,GLU72,THR82 | 30% |
| pol | Integrase | F2WR52 | 6NCJ | Chain A:GLU96,ALA98,GLY94,VAL126,CYS130,TYR99,LEU102,ALA129,ALA128,TRP132,GLN95,THR125,TRP131,LYS127,THR124 | 40% |
| MAP2K1 | Dual specificity mitogen-activated protein kinase kinase 1 | Q02750 | 4AN2 | Chain A:ASN78,LYS97,LEU115,LEU118,VAL127,ILE141,MET143,ASP190,LYS192,ASN195,ASP208,PHE209,GLY210,VAL211,SER212,LEU215,GLY225,THR226 | 60% |
| PRMT3 | Protein arginine N-methyltransferase 3 | O60678 | 4QQN | Chain A:LEU503,ALA467,LYS392,VAL420,VAL387,LEU504,ARG396,VAL501,GLU422,HIS393,LEU424,ASP389,THR466,SER388,VAL390 | 40% |
| INPPL1 | Phosphatidylinositol 3,4,5-trisphosphate 5-phosphatase 2 | O15357 | 6SQU | Chain A:GLU567,THR569,ALA570,ASN573,ASP613,GLN636,LEU639,GLU640,LYS643,LYS645; | 40% |
| coaBC | Coenzyme A biosynthesis bifunctional protein CoaBC | A0QWT2 | 6THC | Chain A:GLU201,ILE209,GLY210,VAL279,ALA280,ASP281,PHE282,ARG283,LEU304,ASN307 | 40% |
| TNF | Tumor necrosis factor | P01375 | 6X82 | Chain A:LYS11,LEU57,ILE58,TYR59,SER60,GLN61,TYR119,LEU120,GLY121,GLY122,VAL123,TYR151,ILE155,ALA156,LEU157 | 60% |
| MTHFD2 | Bifunctional methylenetetrahydrofolate dehydrogenase/cyclohydrolase, mitochondrial | P13995 | 7EHM | Chain A:ASN78,ALA80,SER81,TYR84,GLN132,LEU133,PRO134,GLU141,ARG142,CYS145,ASP155,GLY156,PHE157,HIS158,VAL162,MET165,CYS166,PRO174,THR176,PRO177,LYS203,ASN204,VAL205,MET207,PRO208,MET211,VAL274,ILE276; | 30% |
| KRAS | GTPase Kras | P01116 | 4LUC | Chain A:VAL7,VAL9,GLY10,ALA11,CYS12,GLY13,LYS16,PRO34,THR58,GLY60,GLN61,GLU62,GLU63,ARG68,TYR71,MET72,PHE78,TYR96,GLN99,ILE100 | 50% |
| LDHA | L-lactate dehydrogenase A chain | P00338 | 6SBV | Chain A:ARG169,PHE170,TYR172,LEU173,GLY175,GLU176,ARG177,LEU178,GLY179,VAL180,HIS181,PRO182,GLN233,VAL234,SER237; Chain B:MET63,GLN66,HIS67,SER69,LEU70,THR74,PRO75,LYS76,ILE77,VAL78; Chain C:ARG268 | 40% |
| pfk | ATP-dependent 6-phosphofructokinase | O15648 | 6QU3 | Chain A:GLY197,GLY198,ASP199,GLN202,ARG203,VAL224,PRO225,LYS226,THR227,ASP231,LEU232,SER233,ARG274,ASP275,ILE414,ILE427,ALA430,THR431,SER432,VAL433,ARG434,ARG435 | 30% |
| CHRNA7 | Neuronal acetylcholine receptor subunit alpha-7 | P36544 | 5AFK | Chain E:VAL18,ASP17,ILE19 | 30% |
| rep_1 | Replicase polyprotein 1ab | P0DTD1 | 7AGA | Chain A:PHE8,VAL104,ILE106,GLN107,PRO108,GLY109,GLN110,THR111,ASN151,ASP153,SER158,ILE200,VAL202,ASN203,ASP245,HIS246,ILE249,PHE291,THR292,PRO293,PHE294,ASP295,ARG298 | 40% |
| rep_2 | Replicase polyprotein 1ab | P0DTD1 | 7AXM | Chain A:SER1,ALA210,ILE213,PRO252,LEU253,GLN256,THR257,VAL296,VAL297,ARG298,GLN299,CYS300,SER301,GLY302,VAL303,THR304 | 30% |
| gag-pol | HIV-1 capsid protein | P12497 | 5KGW | Chain A:LEU172,MET178,ALA169,ALA175,GLN168,LYS173,GLU170,HIS171,THR174 | 50% |
| IDH1 | Isocitrate dehydrogenase cytoplasmic | O75874 | 4UMX | Chain A:VAL281 | 40% |

^a^ The classification of allosteric proteins is consistent with ASD 2019.

**REFERENCE**

1. Burley, S.K., Bhikadiya, C., Bi, C., Bittrich, S., Chao, H., Chen, L., Craig, P.A., Crichlow, G.V., Dalenberg, K., Duarte, J.M. *et al.* (2023) RCSB Protein Data Bank (RCSB.org): delivery of experimentally-determined PDB structures alongside one million computed structure models of proteins from artificial intelligence/machine learning. *Nucleic Acids Res*, **51**, D488-D508.

2. Varadi, M., Anyango, S., Deshpande, M., Nair, S., Natassia, C., Yordanova, G., Yuan, D., Stroe, O., Wood, G., Laydon, A. *et al.* (2022) AlphaFold Protein Structure Database: massively expanding the structural coverage of protein-sequence space with high-accuracy models. *Nucleic Acids Res*, **50**, D439-D444.

3. Yang, J., Wang, Y. and Zhang, Y. (2016) ResQ: An Approach to Unified Estimation of B-Factor and Residue-Specific Error in Protein Structure Prediction. *J Mol Biol*, **428**, 693-701.

4. Liebschner, D., Afonine, P.V., Baker, M.L., Bunkoczi, G., Chen, V.B., Croll, T.I., Hintze, B., Hung, L.W., Jain, S., McCoy, A.J. *et al.* (2019) Macromolecular structure determination using X-rays, neutrons and electrons: recent developments in Phenix. *Acta Crystallogr D Struct Biol*, **75**, 861-877.

5. Song, K., Liu, X., Huang, W., Lu, S., Shen, Q., Zhang, L. and Zhang, J. (2017) Improved Method for the Identification and Validation of Allosteric Sites. *J Chem Inf Model*, **57**, 2358-2363.

6. Meyer, M.J., Beltran, J.F., Liang, S., Fragoza, R., Rumack, A., Liang, J., Wei, X. and Yu, H. (2018) Interactome INSIDER: a structural interactome browser for genomic studies. *Nat Methods*, **15**, 107-114.

7. Kim, S., Chen, J., Cheng, T., Gindulyte, A., He, J., He, S., Li, Q., Shoemaker, B.A., Thiessen, P.A., Yu, B. *et al.* (2023) PubChem 2023 update. *Nucleic Acids Res*, **51**, D1373-D1380.

8. Wishart, D.S., Feunang, Y.D., Guo, A.C., Lo, E.J., Marcu, A., Grant, J.R., Sajed, T., Johnson, D., Li, C., Sayeeda, Z. *et al.* (2018) DrugBank 5.0: a major update to the DrugBank database for 2018. *Nucleic Acids Res*, **46**, D1074-D1082.

9. Huang, W., Wang, G., Shen, Q., Liu, X., Lu, S., Geng, L., Huang, Z. and Zhang, J. (2015) ASBench: benchmarking sets for allosteric discovery. *Bioinformatics*, **31**, 2598-2600.
